# Understanding the role of depressive symptoms in academic outcomes: A longitudinal study of college roommates

**DOI:** 10.1371/journal.pone.0286709

**Published:** 2023-06-05

**Authors:** Diane M. Quinn, Amy Canevello, Jennifer K. Crocker

**Affiliations:** 1 Department of Psychological Sciences, University of Connecticut, Storrs, Connecticut, United States of America; 2 Department of Psychology, University of North Carolina, Charlotte, North Carolina, United States of America; 3 Department of Psychology, Ohio State University, Columbus, Ohio, United States of America; University of Glasgow, UNITED KINGDOM

## Abstract

Rising rates of depression among adolescents raise many questions about the role of depressive symptoms in academic outcomes for college students and their roommates. In the current longitudinal study, we follow previously unacquainted roommate dyads over their first year in college (*N* = 245 dyads). We examine the role of depressive symptoms of incoming students and their roommates on their GPAs and class withdrawals (provided by university registrars) at the end of the Fall and Spring semesters. We test contagion between the roommates on both academic outcomes and depressive symptoms over time. Finally, we examine the moderating role of relationship closeness. Whereas students’ own initial levels of depressive symptoms predicted their own lower GPA and more course withdrawals, they did not directly predict the academic outcomes of their roommates. For roommates who form close relationships, there was evidence of contagion of both GPAs and depressive symptoms at the end of Fall and Spring semesters. Finally, a longitudinal path model showed that as depressive symptoms spread from the student to their roommate, the roommate’s GPA decreased. The current work sheds light on a common college experience with implications for the role of interventions to increase the academic and mental health of college students.

## Introduction

Students arriving at college with depressive symptoms tend to achieve lower grade point averages (GPAs) and are more likely to drop out during their first two years at school [1, 2]. Less is known, however, about the implications of students’ depressive symptoms for their roommates’ academic achievement and mental health. Does having a depressed roommate hurt one’s GPA? Are GPAs and depressive symptoms “contagious,” meaning roommates’ GPAs and depressive symptoms become more similar over time? Do these processes depend on the closeness of the roommate relationship? In this study, we probe these questions using data from previously unacquainted roommate dyads, surveyed over the course of their first year in college. We examine the effects of incoming levels of depressive symptoms on GPAs, course withdrawals, and changes in levels of depressive symptoms. We further assess whether any effects depend on roommates’ feelings of closeness with each other.

### Mental health in college

Both the transition to college and the onset of several mental health disorders occur in late adolescence [3]. In recent years, college counseling centers report increasing numbers of students starting college with serious psychological problems [4]. Indeed, research indicates increasing prevalence of depression in the U.S. overall, with the most rapid increase in youth ages 12–17 years and greater increases in adolescent girls compared to boys [5, 6]. According to the American College Health Association’s National College Health Assessment [7], 17% of American college students reported that they had been diagnosed or treated by a professional for depression within the last 12 months. Official diagnosis, however, underrepresents the number of students who may be struggling with depressive symptoms. In the same survey, 68% of students reported they “felt very sad” at some time within the last 12 months; 41% reported they “felt so depressed that it was difficult to function” at some time within the last 12 months; and 12% reported they seriously considered suicide. Thus, many students make the transition to college with mental health issues, including high rates of depressive affect. Depressive symptoms may affect not only these students’ academic outcomes, but potentially their roommates’ as well.

### Depression and academic outcomes

Research on the relationship between depressive symptoms and academic performance in college students finds that increased depression levels are related to lower grades [8–10]. For example, a large longitudinal study measuring undergraduate and graduate students during their first year at university and two years later found that depressive affect at the start of the school year negatively predicted GPA during the first semester of college and positively predicted dropping out of college two years later [2]. No differences were found by gender or race. Likewise, a longitudinal study examining the relationship between depressive affect, GPA, and dropout rates in a sample of Black college students found that higher levels of depressive affect at the start of the academic year predicted both lower first year GPA and higher likelihood of dropping out by the end of the second year [1]. Across multiple studies, the relationship between depressive affect and GPA is not confined to clinically high or diagnosed levels of depression–research shows a pattern of negative relationships even at mild and moderate levels of symptoms [8, 11].

### Social contagion of grade point averages and depressive symptoms

Work on social contagion [12] has shown that attitudes, affect, and behaviors can spread between people and within their close social networks. Because living on campus is believed to increase school engagement [13] and retention [14], many universities require or strongly encourage students to reside on campus for their first year, usually with roommates. A few studies have examined social contagion between roommates’ GPAs. A study at one private college examining all incoming first year students with assigned roommates (i.e., students who did not room with friends from high school) found that roommates’ GPAs were correlated at the end of the first year, controlling for incoming standardized test scores [15]. This effect was replicated in a study of business school students in India [16], which showed that controlling for students’ own GMAT scores, their roommates’ higher GMAT was related to students’ higher GPAs at the end of the year. Similarly, a social network analysis with high school students found that students’ GPAs tended to increase or decrease in line with their closest friends’ GPAs over time [17]. Although these studies show social contagion effects for GPAs, there are very few studies examining college roommate dyads and none of the studies examine whether roommates’ depressive affect can also affect GPAs or GPA contagion.

A separate body of previous research has examined social contagion of depression in roommate relationships. A survey of roommate dyads found that students’ initial levels of depressive symptoms predicted depressive symptoms in their roommates three weeks later [18]. In a longitudinal study examining contagion effects of cognitive vulnerability to depression (i.e., brooding rumination style) and depression among college freshman roommates, results at 3- and 6-months post baseline showed contagion effects of cognitive vulnerability, but no contagion of depressive symptoms [19]. Finally, a large longitudinal study surveyed incoming first year college students right before the start of the school year and again at the end of the school year and examined the extent to which happiness, depression, and anxiety were contagious between roommates [20]. Over the course of the year, the research showed a small contagion effect for anxiety–students whose roommate had high anxiety at the beginning of the year increased in anxiety themselves–but no contagion effects for happiness or depression. Of importance for the current study, this study did not find an effect of roommate depression on feelings of closeness to roommates or how much time the roommates spent together. In sum, existing research on social contagion of roommates’ depression levels is varied, with some studies showing short-term affective contagion and others not.

### Roommate relationship closeness and contagion effects

It may be that the extent to which roommates’ depressive symptoms or grades become similar over time depends on the closeness of the roommate relationship. Whereas previous work has found associations of depression levels between close adolescent friends (for review, [21]) and within large social networks [22], it is difficult to know how much of the statistical relationship is due to homophily–the tendency for people to select and socialize with people similar to themselves. Examining roommates who are unacquainted before being assigned to live together allows for a test of contagion without homophily [23]. In one of the few studies examining contagion and closeness between college roommates, researchers assessed the social contagion of binge drinking during the first year of college between previously unacquainted roommates [23]. Results showed overall peer effects for binge drinking, but also found that these effects were stronger between roommates who considered themselves best friends compared to non-friends. In short, binge drinking was more contagious when roommates were close.

### Current study

In the current study, we examined first year roommate dyads–all of whom were unknown to each other before becoming roommates–from three public universities over the course of their first year in college, surveying them at the start of the year and the end of each semester. We obtained grade reports and course withdrawal data from the university registrars. In this investigation, we use the terms “students” and “roommates” in lieu of “actors” and “partners”, which is the typical language used in investigations of dyadic data. We do this to aid readers who are less familiar with dyadic analyses. Importantly, in this investigation all participants simultaneously hold the role of both “students” and “roommates”. We examine three sets of questions, which we divide into phases in the analyses for greater clarity:

First, in Phase 1, we examined what happens to students’ own academic outcomes when they either enter college with heightened depressive symptoms or are assigned a roommate with heightened depressive symptoms. Further, we tested whether any effects were moderated by roommate closeness. To answer these questions, we analyzed whether depressive symptoms at the start of the year predicted 1) the student’s own GPA for the Fall and Spring semesters; 2) their roommate’s GPA for Fall and Spring; 3) student’s own withdrawal from individual classes (Fall and Spring); and 4) roommate’s withdrawal from individual classes (Fall and Spring). 5) We then examined whether each of these potential associations was moderated by a dyadic measure of roommate closeness.

Second, in Phase 2, we examined whether GPAs, course withdrawals, and depressive affect are contagious between roommates by computing associations between the two roommates’ scores on these measures at each time point (at the start of the year, at end of the Fall semester, and at the end of the Spring semester); and testing whether these relationships are moderated by relationship closeness. If academic outcomes and depressive symptoms are socially contagious, the roommates’ scores should become more correlated over time. If contagion occurs only when roommates are close, the moderation by closeness interaction terms should be significant.

**Fig 1:**
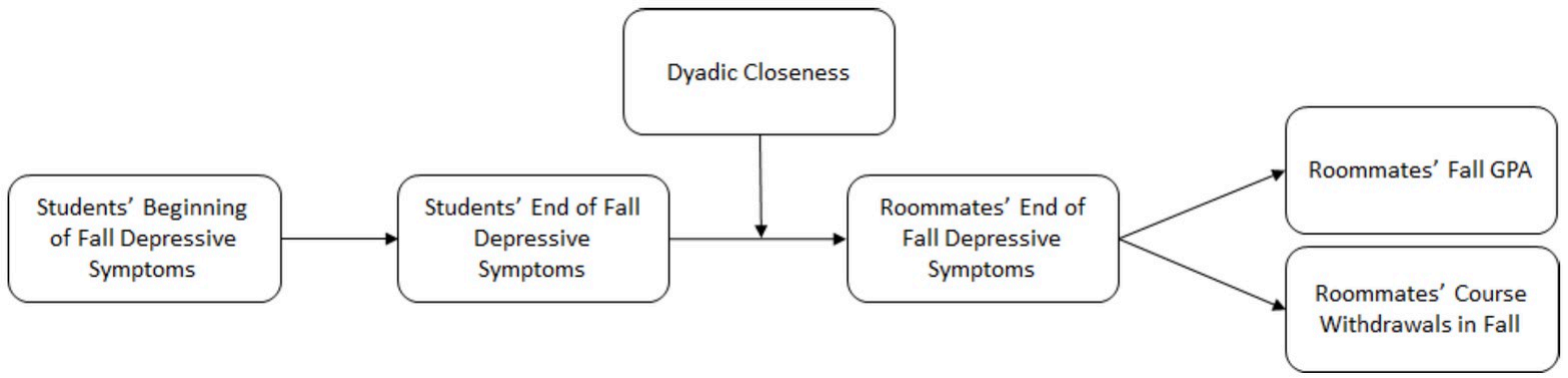
Hypothesized path model.

Third, in Phase 3, we examined whether students’ depressive symptoms early in the academic year ultimately predicted roommates’ fall and spring academic outcomes. As shown in Fig 1, we tested longitudinal associations in which students’ depressive symptoms at the start of the year predict their own depressive symptoms at the end of the Fall semester which in turn predict their roommates’ depressive symptoms (contagion), and whether roommates’ depressive symptoms in turn predict roommates’ own academic outcomes. We included roommate relationship closeness as a moderator of the potential contagion between students’ and their roommates’ depressive symptoms.

Based on previous research, we expected that student’s own depressive symptoms would predict their own academic outcomes, with higher depressive symptoms predicting lower GPAs and more course withdrawals. In addition, based on studies of social contagion between unacquainted roommates, we predicted contagion of academic outcomes and depression, and tested whether this contagion is stronger when the roommate relationship is closer. Because no research has examined how students’ depression might affect their roommates’ academic outcomes, these analyses are exploratory. This study allows an unprecedented look into how depression shapes the academic lives of college students and their roommates over the course of the first year in college.

## Method

### Participants

Participants were 245 same-sex first-semester freshmen roommate dyads (87% female, 13% male;.4% other) at three large public universities in the southeastern, northeastern, and midwestern portions of the United States who were recruited in the fall semesters of 2015, 2016, and 2017. Roommates did not know each other prior to becoming roommates. However, because the universities do assign students to dorms based on reported interests or academic background (e.g., academic honors dorms), assignments are not completely random. All dyads had been living together between 1 and 6 weeks when they began participating (*M* = 2.99 weeks, *SD* = 1.31). Participants ranged in age from 18 to 29 (*M* = 18.19, *SD* = .70) and 82% reported that their race was Caucasian/white, 11% reported their race as Asian American, 8% reported their race was African American/Black, 6% reported their race was Latino/a, 1% reported their race as American Indian/Alaska Native, 1% reported their race as Middle Eastern, and 2% selected other (participants could select multiple racial categories). These data were collected as part of a larger project for which the target number of dyads was 225. A power analysis for dyadic data [24] specifying a moderate correlation between roommates (*r* = .30) and a small correlation of the errors (*r* = .10) suggests that the final sample size of 245 dyads provides power of.80 to detect small effects within and between roommates (*pr* = .13) at *p* = .05 for indistinguishable dyads. All participants provided written consent prior to participation and were paid up to $60 for completing the 3 surveys. Participants completed surveys at four times during the academic year, as well as a multiweek burst, but the current work focuses on 3 timepoints: T1, within the first 6 weeks of the fall semester, T2 at the end of the fall semester, and T3 at the end of the spring semester.

Incoming responses to the surveys were continuously monitored. Participants reporting high levels of distress were contacted by a clinically licensed psychologist and were e-mailed information for counseling resources at their university. Full information on data safety screening is in the supplemental materials. The research and all procedures, including recruitment and safety monitoring, were approved by the Institutional Review Boards at the University of Connecticut (Protocol #H15-081), Ohio State University (Protocol #2015B0170), and the University of North Carolina-Charlotte (Protocol #15-04-31). All authors have complied with the APA ethical standards in treatment of the study participants.

### Measures

At T1, roommates were required to attend an in-person lab session together where they completed scales assessing depressive symptoms, closeness to their roommates, and reported their standardized test scores (i.e., ACT and SAT) within a larger survey. They also gave written permission for the researchers to obtain their fall and spring grades and information about course withdrawals from the University registrars. At T2 and T3 participants completed online surveys; participants again reported their depressive symptoms.

#### Depressive symptoms

We used the Center for Epidemiological Studies Depression Inventory (CES-D; [25]) to assess depressive symptoms at all three time points. The CES-D has been validated in the general population [26] and college samples [27]. Twenty items began with instructions to indicate how often in the past week participants experienced each item, on a scale in which 0 = *rarely or none of the time (less than one day)*, 1 = *some or a little of the time (1–2 days)*, 2 = *occasionally or a moderate amount of time (3–4 days)*, and 3 = *most or all of the time (5–7 days)*. Sample items include “I felt depressed,” “I felt that I could not shake off the blues even with help from my family or friends,” and “I felt sad.” This scale had excellent reliability in this sample (α_T1_ = .91, α_T2_ = .93, and α_T3_ = .94).

Possible scores on the CES-D range from 0 to 60; scores ranged from 0 to 51 at T1, from 0 to 55 at T2, and from 0 to 53 at T3. A score of 16 or greater identifies those at risk for mild to moderate depression [25] with scores of 27 or greater indicating clinical levels of depressive symptoms [28]. On average, depressive symptoms in this sample were just above the threshold for mild to moderate depression (*M*_*T1*_ = 16.80 (10.64), *M*_*T2*_ = 15.92 (11.51), *M*_*T1*_ = 17.18 (12.43)). Just under half (47%) of participants in this sample (*N* = 228) met threshold for risk of mild to moderate depression at T1; eighteen percent (*N* = 87) met threshold for clinical levels of depressive symptoms at T1. At T3, the same percentage of participants met threshold for risk of mild to moderate depression (47%, *N* = 203); twenty-three percent (*N* = 97) met threshold for clinical levels of depressive symptoms.

#### Dyadic closeness

At T1, participants reported their feelings of closeness to their roommates by responding to three items: “How close do you feel to your roommate?”, “Relative to all your other relationships (both same and opposite sex), how would you characterize your relationship with your roommate?”, and “Relative to what you know about other people’s roommate relationships, how would you characterize your relationship with your roommate?” Items were rated on a scale from 1 (*not at all/not as close as others*) to 5 (*extremely/much closer than others*) and demonstrated adequate reliability in this sample (α = .89). Students’ and roommates’ feelings of closeness were correlated (*r* = .55), so we averaged them to create an indicator of dyadic closeness between roommates (α = .88).

#### Standardized test scores

Students reported their verbal and quantitative ACT and SAT scores during the initial lab session. Possible raw ACT scores ranged from 1 to 36, *M* = 28.69, *SD* = 3.96. Possible raw SAT subscores ranged from 200 to 800, *M* = 616.83, *SD* = 76.22. We standardized each reported score and averaged across individual students’ scores to create a standardized test score (α = .86).

#### GPA

Students’ grades were collected from university registrars at the end of the fall and spring semesters. Possible GPA ranged from 0 to 4.0 (*M*_Fall_ = 3.19 *SD*_Fall_ = .75, *M*_Spring_ = 3.17, *SD*_Fall_ = .85).

#### Course withdrawals

University registrars also reported the courses from which students had withdrawn in the fall and spring semesters. In the fall semester, 9% of participants withdrew from at least one course; of those, less than 2% withdrew from two or more courses. In the spring semester, 13% of participants withdrew from at least one courses; of those, 2% withdrew from two or more courses. Because of the relative infrequency of withdrawing from multiple courses in both semesters, we created variables for fall and spring that were coded such that 1 indicated that students had withdrawn from a least one course during the semester and 0 indicated that students had not withdrawn from any courses during the semester.

## Results

### Overview of analyses

Analyses were conducted in phases that matched our three larger aims. In Phase 1, we examined whether students’ and roommates’ T1 depressive symptoms were associated with students’ fall (T2) and spring (T3) GPAs. Next, we tested whether students’ and roommates’ T1 depressive symptoms were related to students’ withdrawal from courses during the fall and spring semesters. Finally, we tested whether these associations were moderated by dyadic closeness.

Phase 2 analyses examined contagion between students and their roommates of GPAs, course withdrawals, and depressive symptoms each semester. Again, we tested whether contagion effects were moderated by dyadic closeness.

In Phase 3, we tested the indirect effects in path models in which students’ depressive symptoms early in the academic year ultimately predicted roommates’ fall and spring academic outcomes. Specifically, we tested one model (Fig 1) in which students’ T1 depressive symptoms predicted their greater T2 depressive symptoms, which in turn predicted roommates’ T2 depressive symptoms, leading to roommates’ fall GPA and course withdrawals. We also tested whether closeness moderated the paths from students’ T2 depressive symptoms to roommates’ T2 depressive symptoms. In a second parallel model, we examined whether students’ T1 depressive symptoms predicted their greater T3 depressive symptoms, which then predicted roommates’ T3 depressive symptoms, leading to roommates’ spring GPA and course withdrawals. We tested whether closeness moderated the path from students’ T3 depressive symptoms to roommates’ T3 depressive symptoms.

### General analytic strategy

In these data, individuals were nested within roommate pairs, so analyses needed to account for the nonindependence of individuals within dyads. For all analyses, we structured the data so that each dyad was represented by two lines of data, allowing each participant within a dyad to represent both a student and a roommate (see [29], for a sample arrangement of data). We standardized all variables to obtain standardized estimates. For all analyses predicting GPA, we controlled for standardized test scores. For all tests of moderation, we added the main effect of the moderator and its interaction with the primary predictor to the original analyses; each moderator was tested in a separate analysis. All simple slopes analyses tested slopes at 1 SD above and below the mean of the moderator. Table 1 shows the means, standard deviations, and within-person and interpersonal (i.e., student-roommate) intraclass correlations [30] for all variables at the beginning of the semester.

**Table 1:** Means, standard deviations, and within-person and interpersonal intraclass correlations for all study variables.

	1.	2.	3.	4.	5.	6.	7.	8.	9.	*M* (*SD*)
1. Depressive Symptoms (T1)	**.02**	**-.13** **	**.09**	**-.10** *	**-.02**	**.01**	**-.05**	**.01**	**-.09**	16.80 (10.64)
2. Dyadic Closeness (T1)	-.13**	**—**	**.00**	**.09** *	**.01**	**.05**	**-.01**	**-.06**	**.01**	3.24 (.90)
3. Depressive Symptoms (T2)	.59***	.00	**.12** *	**-.09**	**.00**	**.08**	**-.10** *	**-.00**	**-.05**	15.92 (11.51)
4. Fall GPA	-.20***	.09*	-.21***	**.25** ***	**-.10** *	**.01**	**.23** ***	**-.01**	**.09**	3.19 (.75)
5. Fall Withdrawals	.14**	.01	.08	-.25***	**.11** *	**-.01**	**-.08**	**.06**	**-.06**	.09 (.28)
6. Depressive Symptoms (T3)	.49***	.05	.56***	-.11*	-.03	**.12** *	**-.02**	**.03**	**-.01**	17.18 (12.43)
7. Spring GPA	-.17***	-.01	-.16**	.67***	-.18***	-.16**	**.23** ***	**-.01**	**.12** *	3.17 (.85)
8. Spring Withdrawals	.14**	-.06	.06	-.24***	.01	.12*	-.21***	**-.03**	**-.07**	.13 (.34)
9. Standardized Test Scores	-.05	.01	-.08	.21***	-.11*	.00	.20***	-.07	**.39** ***	.01 (.84)

*Note*: *N*_beginning of fall_ = 490; *N*_3 weeks_ = 453; *N*_end of fall_ = 431; *N*_end of spring_ = 429.

* p < .05,

** p < .01,

*** p < .001.

Scores on and above the diagonal (in grey) are interpersonal intraclass correlations (i.e., between students and roommates). Scores below the diagonal are within-person intraclass correlations.

For analyses predicting depression and GPA (i.e., continuous variables), we conducted analyses using the MIXED command in SPSS (Version 27; [29, 31, 32]). As recommended by Kenny and colleagues [32], coefficients were derived from a random-coefficients model using restricted maximum-likelihood estimation. In tests of all Phase 1 analyses using MIXED models, we specified a compound symmetry covariance structure to accommodate indistinguishable dyads. In all Phase 2 analyses using MIXED models, the models failed to converge with this covariance structure; we specified a variance components covariance structure in these analyses. For analyses predicting course withdrawals, which was coded as a binary variable, we used generalized estimating equations (GEE) methodology using the GENLIN command in SPSS [33]. In specifying these models, we followed recommendations from Loeys and colleagues [33] by specifying a binomial distribution and logit-link function, indicating an unstructured working correlation matrix, and requesting robust standard errors with associated Wald tests. Detailed reports of findings can be found in S1 and S2 Tables. Phase 3 analyses focused on testing conditional indirect effects using structural equation modeling and followed the procedures described by Ledermann and colleagues [34], Macho and Ledermann [35] and Olsen and Kenny [36] because these approaches are well suited to the assessment of mediation in dyadic data with indistinguishable dyads. We tested phantom models in AMOS (version 28) using 5,000 bootstrapped samples and reported the bias-corrected bootstrap 95% CI. Across models, we modeled coefficients for students’ and roommates’; because dyad members were indistinguishable, we constrained all parameters to be equal for students and roommates.

### Missing data

Attrition in this study was relatively low; of the 490 participants who began the study, 431 (88%) completed the T2 survey, and 429 (87.6%) completed the T3 survey at the end of the spring semester. Students’ depressive symptoms at T1 were not significantly related to attrition at T2 (Exp(.14) = 1.15, 95% CI for Exp(B) [.91, 1.46], *p* = .255), but were associated with an increased likelihood of attrition at T3 (i.e., a one standard deviation [SD] increase in T1 depressive symptoms increased the likelihood of attrition at T3 by 32% (Exp(.28) = 1.32, 95% CI for Exp(B) [1.08, 1.61], *p* = .006). Closeness of roommates at T1 was not significantly related to attrition at T2 (Exp(-.18) = .84, 95% CI for Exp(B) [.58, 1.22], *p* = .358) or T3 (Exp(-.13) = .88, 95% CI for Exp(B) [.60, 1.30], *p* = .514).

Attrition contributed to missing values for depressive symptoms at T2 (12%) and T3 (12.4%). Additionally, a similar proportion did not report standardized test scores (12.2%). Missingness was low for fall and spring GPAs (2.9% and 6.1%, respectively) and fall and spring course withdrawals (2.4% and 4.1%, respectively). There were no missing data for T1 depressive symptoms or dyadic closeness. Missing data were treated using listwise deletion in Phase 1 and 2 analyses. Because Phase 3 analyses required complete data (i.e., no missing observations) in order to accommodate bootstrapping to test indirect effects, we treated missing data using Bayesian imputation.

### Phase 1 analyses

#### Students’ and roommates’ T1 depressive symptoms predicting students’ fall and spring GPAs

We tested whether students’ and roommates’ T1 depressive symptoms predicted students’ fall and spring GPAs. In two separate analyses, we regressed students’ fall and spring GPAs on students’ and roommates’ T1 depressive symptoms and students’ standardized test scores. Students’ T1 depressive symptoms predicted their lower GPAs in both semesters (fall: b = -.19, t(410.67) = -3.90, 95% CI [-.28, -.09], *p* < .001; spring: b = -.14, t(398.95) = -3.01, 95% CI [-.24, -.05], *p* = .003). Students whose T1 depressive symptoms were at 1 SD below the sample mean (a score of 6.16, which is well below threshold for risk of clinical depression) had a predicted fall GPA of 3.36 and spring GPA of 3.33, whereas students whose T1 depressive symptoms were at 1 SD above the mean (a score of 27.44, which is just above threshold for a diagnosis of clinical depression), had a predicted GPA of 3.07 in fall and 3.03 in spring. Roommates’ T1 depressive symptoms were not significantly related to students’ GPAs in either semester (fall: b = -.09, t(409.58) = -1.81, 95% CI [-.18,.01], *p* = .072; spring: b = -.03, t(395.78) = -.67, 95% CI [-.12,.06], *p* = .501). Dyadic closeness did not moderate any of these associations (*p*s ≥.089, *pseudo ΔR*^*2*^ ≤.005).

#### Students’ and roommates’ T1 depressive symptoms predicting students’ course withdrawals

Next, we tested whether students’ and roommates’ T1 depressive symptoms predicted students’ course withdrawals during the fall and spring semesters. When we regressed students’ fall and spring withdrawals on students’ and roommates’ depressive symptoms at T1 in two separate analyses, students’ T1 depressive symptoms predicted their greater likelihood of withdrawing from at least one course both semesters. Specifically, a one SD increase in students’ T1 depressive symptoms increased the odds of their withdrawing from a class by 60% in the fall semester (Exp(.47) = 1.60, 95% CI for Exp(B) [1.15, 2.22], *p* = .005) and by 46% in the spring semester (Exp(.38) = 1.46, 95% CI for Exp(B) [1.14, 1.86], *p* = .002). Roommates’ T1 depressive symptoms were not significantly related to the likelihood of students’ course withdrawals in either semester (fall: Exp(-.08) = .92, 95% CI for Exp(B) [.69, 1.23], *p* = .571; spring: Exp(.01) = .94, 95% CI for Exp(B) [.77, 1.33], *p* = .936). Dyadic closeness did not moderate any of these associations (*p*s ≥.286).

These findings replicate previous research demonstrating that students’ depressive symptoms are associated with their own lowered GPAs and a greater likelihood of withdrawing from at least one course. Roommates’ depressive symptoms did not predict students’ academic performance. These associations did not depend on levels of closeness between roommates.

### Phase 2

#### Social contagion of GPA

We tested the contagion of GPA during the fall and spring semesters. In these analyses, we controlled for both people’s standardized test scores. When we regressed students’ fall GPA on roommates’ fall GPA and students’ and roommates’ standardized test scores, roommates’ fall GPA positively predicted students’ fall GPA (b = .19, t(333.89) = 3.79, 95% CI [.09,.28], *p* < .001). When roommates’ fall GPAs were lower (i.e., at 1 SD below the sample mean; a GPA of 2.44), the predicted value of students’ fall GPAs was 3.09, whereas when roommates’ fall GPAs were higher (i.e., at 1 SD above the sample mean; a GPA of 3.94), the predicted value of students’ fall GPAs was 3.37. In a similar analysis of GPA contagion in the spring semester, roommates’ spring GPA positively predicted students’ spring GPA (b = .20, t(278.48) = 4.22, 95% CI [.11,.30], *p* < .001). When roommates’ spring GPAs were lower (a GPA of 2.32), the predicted value of students’ spring GPAs was 3.07, whereas when roommates’ spring GPAs were higher (a GPA of 3.94), the predicted value of students’ spring GPAs was 3.41.

Because in Phase 1 analyses, students’ T1 depressive symptom were related to their fall and spring GPAs and roommates’ T1 depressive symptoms were marginally related to students’ fall GPA, we conducted these analyses a second time, including students’ and roommates’ T1 depressive symptoms as additional covariates. Results remained unchanged: roommates’ and students’ GPAs were positively related in fall (b = .16, t(335.46) = 3.20, 95% CI [.06,.26], *p* = .001) and spring (b = .19, t(286.83) = 3.97, 95% CI [.10,.29], *p* < .001).

**Fig 2:**
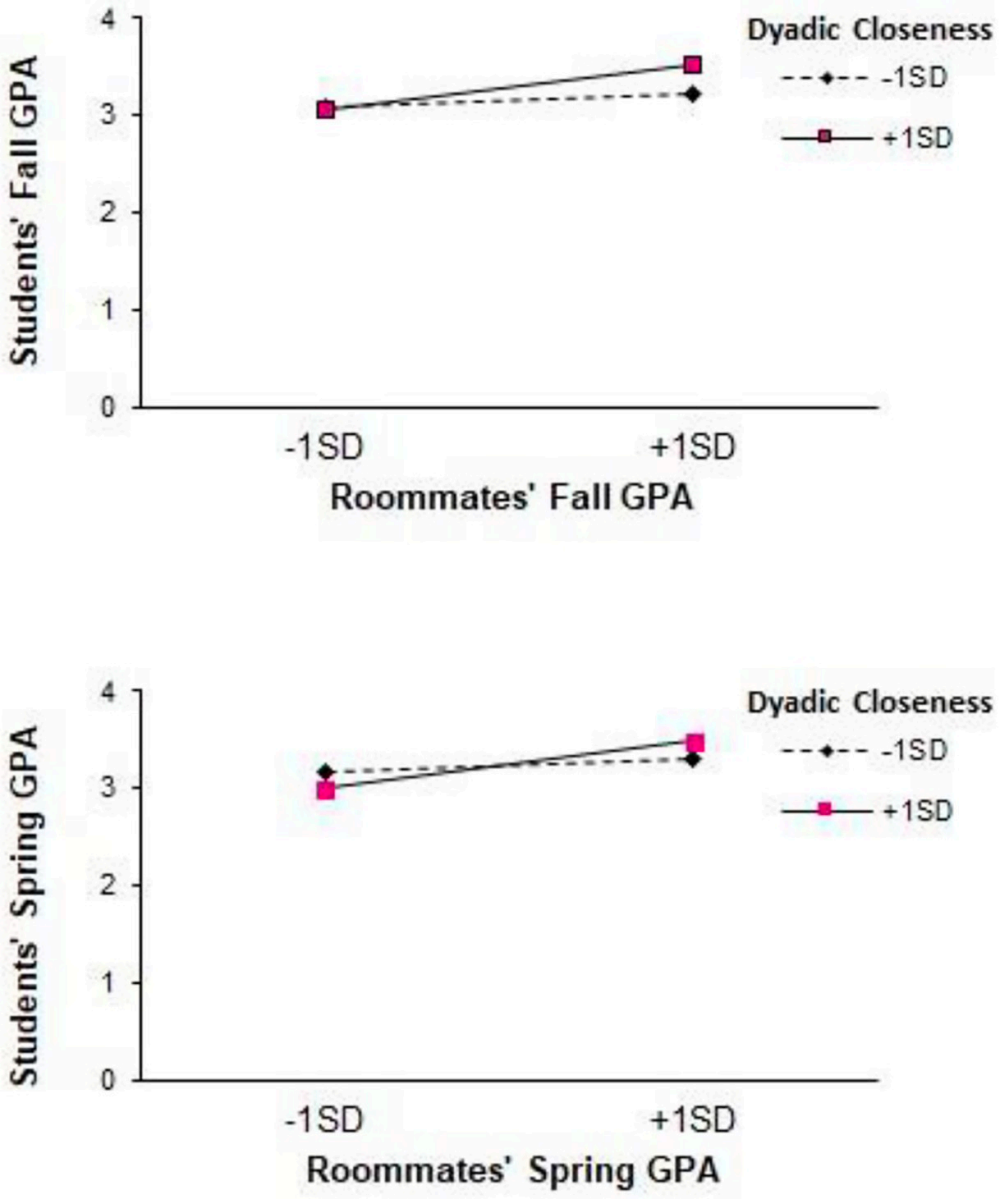
GPA Contagion in the Fall (upper panel) and Spring (lower panel) Moderated by Dyadic Closeness. **Note**. These figures depict the simple slopes for GPA contagion at 1 standard deviation above and below the mean of T1 dyadic closeness. The endpoints for these slopes are plotted at 1 standard deviation above and below the means for roommates’ GPAs in fall (upper panel) and spring (lower panel).

GPA contagion was moderated in the fall and spring by dyadic closeness (fall: *p* = .028, *pseudo ΔR*^*2*^ = .01, see upper panel of Fig 2; spring: *p* = .016, *pseudo ΔR*^*2*^ = .02; see lower panel of Fig 2). Simple slopes analyses suggested that roommates’ and students’ GPAs were positively related when dyadic closeness at T1 was one standard deviation above the mean (fall: b = .30, *t*(329.83) = 4.08, 95% CI [.16,.45], *p* < .001; spring: b = .29, *t*(258.50) = 4.84, 95% CI [.17,.40], *p* < .001), but were not significantly related when T1 dyadic closeness was one standard deviation below the mean (fall: b = .10, *t*(317.69) = 1.76, 95% CI [-.01,.22], *p* = .080; spring: b = .08, *t*(296.84) = 1.08, 95% CI [-.06,.22], *p* = .282). Johnson-Neyman analyses conducted using the method described by Preacher and colleagues [37] suggest that contagion of GPA in the fall occurred when T1 dyadic closeness was at or greater than.91 standard deviation below the mean; contagion of GPA in the spring occurred when T1 dyadic closeness was at or greater than.66 standard deviation below the mean. That is, contagion of GPA in the fall was significant when dyads reported mutual closeness of 2.33 and higher at T1; contagion of GPA in the spring was significant when dyads reported mutual closeness of 2.58 and higher at T1.

#### Contagion of course withdrawals

We tested contagion of course withdrawals during the fall and spring semesters. When we regressed students’ fall and spring withdrawals on roommates’ fall and spring withdrawals, respectively, in separate analyses, the GEE models failed to converge suggesting that the specified models were inappropriate for the data, likely because of the relatively low intraclass correlations for withdrawals in fall and spring (see Table 1).

We simplified the analyses by conducting logistic regressions. When roommates withdrew from at least one course in the fall semester, the likelihood that students also withdrew from at least one course that semester was almost three times higher (Exp(1.00) = 2.73, 95% Wald CI for Exp(B) [1.17, 6.36], *p* = .020). There was no evidence of contagion of withdrawal from classes during the spring semester: roommates’ course withdrawal during the spring semester was not significantly related to students’ likelihood of withdrawing from at least one course in the spring semester (Exp(-.33) = .72, 95% Wald CI for Exp(B) [.30, 1.76], *p* = .471).

Because in Phase 1 analyses, students’ T1 depressive symptoms were related to their fall and spring course withdrawals, we conducted these analyses a second time, including students’ T1 depressive symptoms as a covariate. (Roommates’ T1 depressive symptoms were not significantly related to students’ withdrawals in either semester so we did not include it as a covariate.) Results remained unchanged: roommates’ and students’ withdrawal from at least one class were positively related in fall (Exp(1.10) = 3.00, 95% Wald CI for Exp(B) [1.26, 7.10], *p* = .013) but not in spring (Exp(-.34) = .71, 95% Wald CI for Exp(B) [.29, 1.75], *p* = .461). Dyadic closeness did not moderate contagion of withdrawals in the fall (*p* = .464, *pseudo ΔR*^*2*^ = .001) or spring (*p* = .439, *pseudo ΔR*^*2*^ = .002).

#### Social contagion of depressive symptoms

We examined the contagion of T1, T2, and T3 depressive symptoms. In three separate models, we regressed students’ depressive symptoms onto roommates’ depressive symptoms within T1, T2, and T3. As would be expected given unacquainted dyads, roommates’ and students’ depressive symptoms were not significantly related at T1 (b = .03, *t*(428.83) = .59, 95% CI [-.06,.11], *p* = .553). This association was not moderated by dyadic closeness (*p* = .081, *pseudo ΔR*^*2*^ = .005).

**Fig 3:**
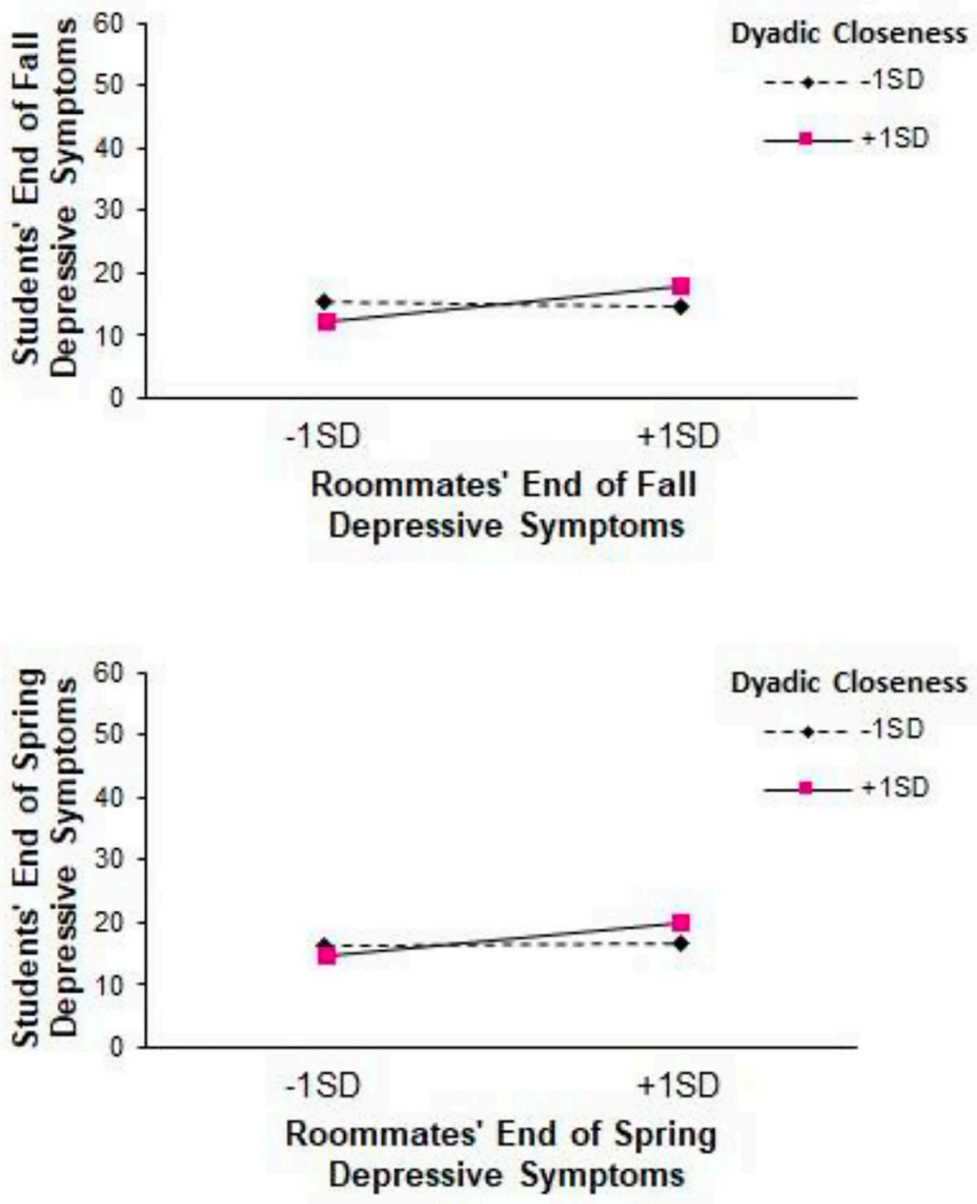
Contagion of Depressive Symptoms at the end of Fall (upper panel) and Spring (lower panel) Moderated by Dyadic Closeness. **Note**. These figures depict the simple slopes for contagion of depressive symptoms at 1 standard deviation above and below the mean of T1 dyadic closeness. The endpoints for these slopes are plotted at 1 standard deviation above and below the means for roommates’ depressive symptoms in fall (upper panel) and spring (lower panel).

Depressive symptoms were contagious at T2. Roommates’ T2 depressive symptoms predicted students’ T2 depressive symptoms (b = .12, *t*(334.17) = 2.61, 95% CI [.03,.21], *p* = .009). This association was moderated by dyadic closeness (*p* = .007, *pseudo ΔR*^*2*^ = .02; see the upper panel of Fig 3). Simple slopes analyses suggested that contagion of depressive symptoms was significant when dyadic closeness at T1 was one standard deviation above the mean (b = .25, *t*(326.90) = 3.77, 95% CI [.12,.38], *p* < .001) but not when dyadic closeness at T1 was one standard deviation below the mean (b = -.03, *t*(346.89) = -.44, 95% CI [-.18,.11], *p* = .661). Johnson-Neyman analyses suggest that contagion of depressive symptoms in the fall occurred when T1 dyadic closeness was at or greater than.13 standard deviation below the mean. That is, contagion of depressive symptoms in the fall was significant when dyads reported mutual closeness of 3.11 and higher at T1.

Depressive symptoms were also contagious at T3. Roommates’ T3 depressive symptoms predicted students’ greater T3 depressive symptoms (b = .12, *t*(380.35) = 2.48, 95% CI [.03,.22], *p* = .014). Again, this association was moderated by dyadic closeness (*p* = .034, *pseudo ΔR*^*2*^ < .01; see lower panel of Fig 3). Simple slopes analyses suggested that contagion of T3 depressive symptoms was significant when dyadic closeness at T1 was one standard deviation above the mean (b = .22, *t*(369.61) = 3.24, 95% CI [.09,.35], *p* = .001) but not when T1 dyadic closeness was at one standard deviation below the mean(b = .01, *t*(370.41) = .14, 95% CI [-.13,.15], *p* = .887). Johnson-Neyman analyses suggest that contagion of depressive symptoms in the spring occurred when T1 dyadic closeness was at or greater than.20 standard deviation below the mean. Thus, contagion of depressive symptoms was significant when dyads reported mutual closeness of 2.58 and higher at T1.

At the end of both semesters, when roommates’ depressive symptoms were lower (i.e., at 1 SD below the sample mean; a score of 4.41 in fall and 4.75 in spring), the predicted values of students’ depressive symptoms were 13.66 and 15.41 at the end of the fall and spring semesters, respectively. Notable, these scores are just below the threshold for risk of mild to moderate depression. However, when roommates’ depressive symptoms were higher (i.e., at 1 SD above the sample mean; a score of 27.43 in fall and 29.61 in spring), the predicted values of students’ depressive symptoms were 16.46 and 18.39 at the end of fall and spring, respectively. These scores are just above the threshold for risk of mild to moderate depression.

These findings provide evidence that GPA and depressive symptoms are contagious between roommates across the academic year and that contagion is strongest when roommates feel closer to each other at the beginning of the academic year. The data also provide evidence of contagion for the likelihood of withdrawing from at least one class in the fall semester, but not in the spring and this association does not depend on roommate closeness in either semester.

### Phase 3

#### Contagion of depressive symptoms predicting roommates’ GPA and withdrawals

**Fig 4:**
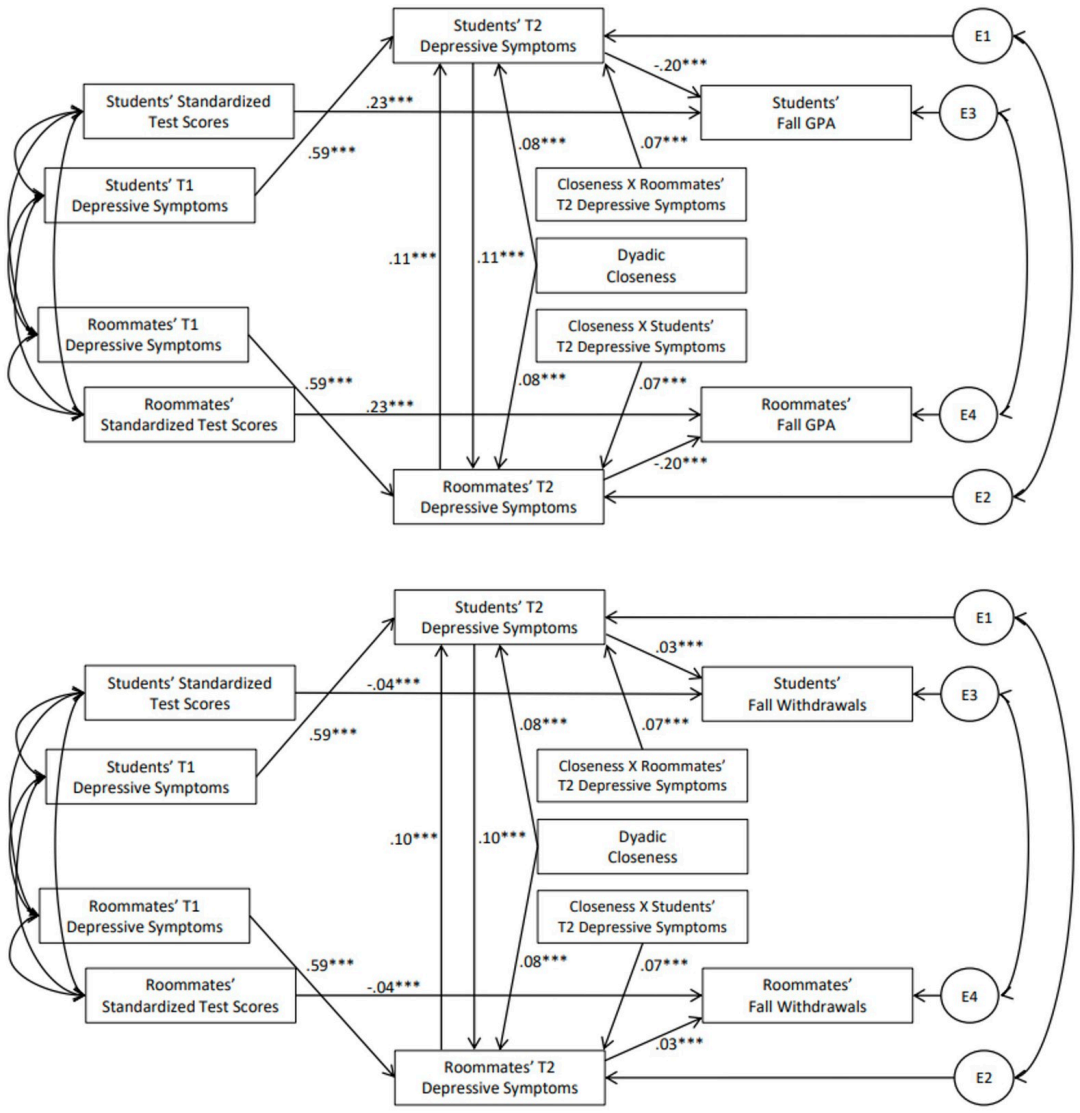
Path coefficients for conditional mediation models predicting roommates’ GPA and course withdrawals in fall. **Note**. All coefficients represent standardized effects; E indicates an error term. ***p < .001.

**Fig 5:**
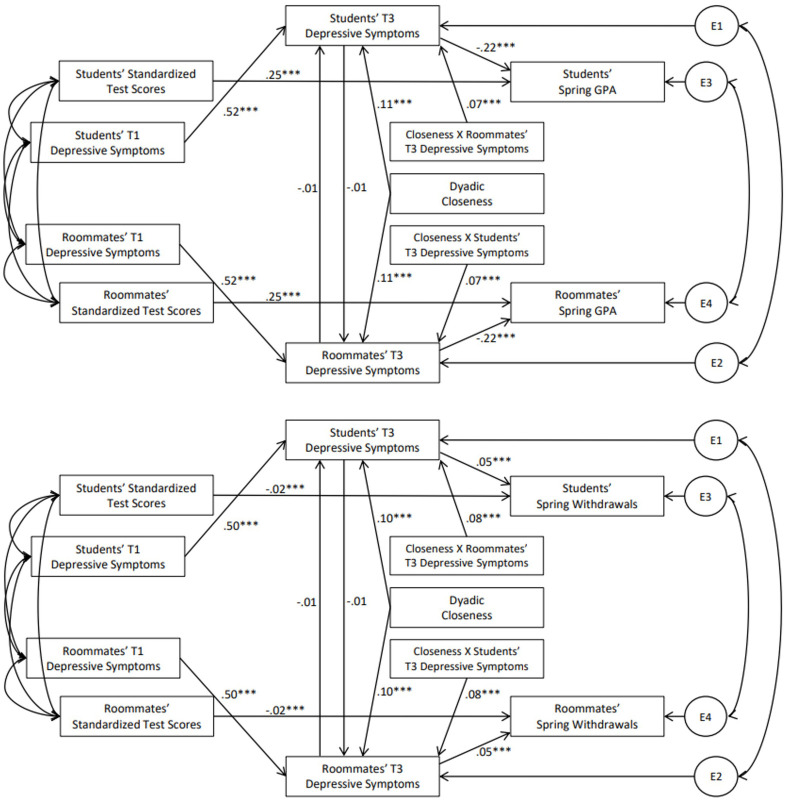
Path coefficients for conditional mediation models predicting roommates’ gpa and course withdrawals in spring. **Note**. All coefficients represent standardized effects; E indicates an error term. ***p < .001.

Taken together, the findings thus far suggest that students’ T1 depressive symptoms may have downstream consequences for roommates’ T2 or T3 academic outcomes through contagion of depression. Thus, we examined whether students’ T1 depressive symptoms and the contagion of depressive symptoms between roommates at T2 and T3 predicted roommates’ GPA and course withdrawals in the fall and spring, respectively. Specifically, we tested two path models. The first model focused on contagion and outcomes at T2, testing whether students’ T1 depressive symptoms predicted their own T2 depressive symptoms, which predicted roommates’ T2 depressive symptoms, which in turn predicted roommates’ GPA and course withdrawals in the fall semester. The path coefficients for these models appear in Fig 4. The second set of analyses tested a similar model of contagion and outcomes at T3. Specifically, the second model tested whether students’ T1 depressive symptoms predicted students’ own T3 depressive symptoms, which predicted roommates’ T3 depressive symptoms, which in turn predicted roommates’ GPA and withdrawals in the spring. In each model, we tested whether contagion of depressive symptoms was moderated by dyadic closeness. The path coefficients for these models appear in Fig 5.

In the model predicting roommates’ fall GPA, students’ T1 depressive symptoms predicted students’ T2 depressive symptoms (standardized estimate = .59, 95% CI [.57,.62], *p* < .001). The path from students’ T2 depressive symptoms to roommates’ T2 depressive symptoms was moderated by dyadic closeness (effect = .07, 95% CI [.05,.10], *p* < .001). Roommates’ T2 depressive symptoms, in turn, predicted their lower fall GPA (standardized estimate = -.20, 95% CI [-.22, -.17], *p* < .001). The index of conditional mediation was significant (effect = -.01, 95% CI [-.013, -.005], *p* < .001), such that the indirect effect was significant for dyads who were higher in closeness (effect = -.02, 95% CI [-.03, -.02], *p* < .001) but not for dyads who were lower in closeness (effect = -.00, 95% CI [-.01,.00], *p* = .161). That is, students’ T1 depressive symptoms indirectly predicted roommates’ GPA in the fall semester, but only when closeness was higher.

In the model predicting roommates’ fall withdrawals, students’ T1 depressive symptoms predicted students’ T2 depressive symptoms (standardized estimate = .59, 95% CI [.57,.62], *p* < .001). The path from students’ T2 depressive symptoms and roommates’ T2 depressive symptoms was moderated by dyadic closeness (effect = .07, 95% CI [.05,.10], *p* < .001). Roommates’ T2 depressive symptoms, in turn, predicted their increased fall course withdrawals (standardized estimate = .03, 95% CI [.02,.04], *p* < .001). The index of conditional mediation was significant (effect = .001, 95% CI [.001,.002], *p* < .001), such that the indirect effect was significant for dyads who were higher in closeness (effect = .003, 95% CI [.002,.004], *p* < .001) but not for dyads who were lower in closeness (effect = .001, 95% CI [.000,.001], *p* = .167). That is, students’ T1 depressive symptoms indirectly predicted roommates’ course withdrawals in the fall semester, but again only when closeness was higher.

In the model predicting roommates’ spring GPA, students’ T1 depressive symptoms predicted students’ T3 depressive symptoms (standardized estimate = .52, 95% CI [.50,.54], *p* < .001). The path from students’ T3 depressive symptoms and roommates’ T3 depressive symptoms was moderated by dyadic closeness (effect = .07, 95% CI [.04,.11], *p* < .001). Roommates’ T3 depressive symptoms, in turn, predicted their lower spring GPA (standardized estimate = -.22, 95% CI [-.25, -.19], *p* < .001). The index of conditional mediation was significant (effect = -.01, 95% CI [-.012, -.005], *p* < .001); the indirect effect was negative and significant for dyads who were higher in closeness (effect = -.01, 95% CI [-.014, -.00], *p* = .040) but positive and significant for dyads who were lower in closeness (effect = .01, 95% CI [.004,.016], *p* = .001). That is, students’ T1 depressive symptoms indirectly predicted roommates’ GPA in the spring. The direction of this indirect association depended on closeness. In closer dyads, students’ higher depressive symptoms indirectly predicted roommates’ lower GPA, but in less close dyads, students’ higher depressive symptoms indirectly predicted roommates’ higher GPA.

In the model predicting roommates’ spring withdrawals, students’ T1 depressive symptoms predicted students’ greater T3 depressive symptoms (standardized estimate = .50, 95% CI [.48,.53, *p* < .001). The path from students’ T3 depressive symptoms and roommates’ T2 depressive symptoms was moderated by dyadic closeness (effect = .08, 95% CI [.04,.11], *p* < .001). Roommates’ T3 depressive symptoms, in turn, predicted their increased spring withdrawals (standardized estimate = .05, 95% CI [.04,.06], *p* < .001). The index of conditional mediation was significant (effect = .002, 95% CI [.001,.003], *p* < .001), such that the indirect effect was positive and significant for dyads who were higher in closeness (effect = .002, 95% CI [.000,.003], *p* = .026) but negative and significant for dyads who were lower in closeness (effect = -.002, 95% CI [-.003, -.001], *p* = .001). That is, students’ T1 depressive symptoms indirectly predicted roommates’ higher course withdrawal in the spring. The direction of this indirect association depended on closeness. In closer dyads, students’ higher depressive symptoms indirectly predicted roommates’ higher course withdrawal, but in less close dyads, students’ higher depressive symptoms indirectly predicted roommates’ lower course withdrawal.

## Discussion

Many students begin college with mental health concerns, including high rates of depressive affect [7]. Previous research finds that depression negatively affects these students’ academic outcomes [1, 2]. Although many colleges and universities encourage first year students to live on campus, usually with one or more roommates, little is known about whether and how one student’s depressive symptoms at the start of college affects their roommate’s outcomes over the first year of college. Students, parents, and educators may be concerned about the potential effects of having a roommate with relatively high levels of depressive symptoms. The present investigation addressed this issue in a longitudinal study of roommate dyads.

We examined three broad issues. First, we examined whether students’ own or their roommates’ depressive symptoms at the start of college predict their academic outcomes in the first year of college. Replicating previous work, we found that students who entered college with more symptoms of depression tended to have lower GPAs at the end of the first and second semesters of college. They were also more likely to have withdrawn from at least one class. These findings reinforce previous research showing that depressive symptoms place students at risk of poor academic outcomes. However, having a roommate with heightened depressive symptoms did not directly predict student’s own GPA or course withdrawals, even in relatively close roommate relationships. The absence of a direct effect of a roommate’s depressive symptoms on students’ own academic outcomes provides some reassurance that having a depressed roommate need not have negative effects on students’ academic progress.

Second, we examined whether GPAs, course withdrawals, and depression are socially contagious between roommates. That is, we tested whether students’ academic outcomes are linked to their roommates’, controlling for any preexisting similarities in their SAT and ACT scores. Indeed, we found evidence of social contagion. Students’ and roommates’ fall and spring GPAs correlated with each other, even controlling for standardized test scores. Furthermore, this contagion effect depended on the closeness of the relationship. In both semesters, when students and their roommates were closer, their GPAs were more strongly linked. For less close dyads, however, GPAs were not related. Course withdrawals show evidence of contagion in the fall semester, but not spring semester, and did not depend on the closeness of the relationship in either semester.

Depressive symptoms also showed evidence of social contagion. As would be expected given that students did not know each other prior to becoming roommates, their depression levels at the start of the school year were uncorrelated. However, by the end of the fall semester and continuing at the end of the spring semester, roommates’ depression levels were correlated. Again, this association depended on the closeness of the relationship. In more close dyads, levels of depressive symptoms were related. In less close dyads, depression levels were unrelated. These findings suggest that roommates influence each other’s academic and mental health outcomes, particularly when the roommates form close friendship bonds.

Although we focus on the negative effects of increased depressive symptoms, these are correlations, so just as higher levels of depression can be contagious, so can lower levels. Indeed, the top panel of Fig 3 shows exactly this pattern: For roommates who are close, when the roommate has low levels of the depressive symptoms, the student’s levels are also lower; when the roommate has higher levels of depressive symptoms, the student’s are also higher. Likewise, having a close roommate with a high GPA is related to a student’s GPA being higher.

Close relationships influence how people feel and behave. This study shows that over the course of the year school year, depressive affect can be contagious between roommates and, thus, it may behoove schools to consider offering support to both roommates when one roommate presents with depression. Although being close to a depressed roommate predicts slightly higher depression levels– 3 points on average–it is important not to lose sight of the larger issue of the sheer number of students starting college with elevated depression symptoms [7].

Third, we examined whether these two general findings—negative effects of depressive symptoms on students’ own academic outcomes, and contagion of depressive symptoms between roommates—constitute a path through which a student’s higher depressive symptoms indirectly predict their roommate’s lower grades through contagion of depressive symptoms (See Fig 1). We found evidence for such an indirect association, but only in close relationships. As an example of how this might work, imagine two roommates: Sarah and Janae. Sarah and Janae form a close friendship right from the start of the school year. Sarah starts college with heightened levels of depressive symptoms, often feeling sad and that everything she did was an effort. By the end of the Fall semester, Sarah continues to feel somewhat depressed and these feelings are contagious: Janae now experiences more frequent depressive symptoms as well. Janae’s increased depressive symptoms predict her lower GPA at the end of the semester.

In less close relationships, the indirect effect of roommates’ depressive symptoms on students’ GPA and withdrawal from classes was not significant in the fall semester, and was in the opposite direction in the spring semester. That is, by the end of the spring semester, roommates’ depressive symptoms indirectly predicted students’ higher GPA and lower withdrawals. This finding raises the possibility that distancing oneself from a roommate with high levels of depressive symptoms may be self-protective, at least when academic outcomes are considered.

### Implications

This research contributes to existing research on depression in college students. Although evidence of contagion of depressive symptoms is not new [22, 38], effects are often inconsistent or include modifying factors. For example, depression seems to be more contagious between women than men and between peers rather than family members [22]; among adolescents, initial levels of social anxiety, and the popularity and status of peers can be modifying factors [21]. Our findings point to the dyadic closeness of relationships as a crucial moderator of the social contagion of depressive symptoms.

Understanding the role of relationship closeness in contagion may allow researchers to gain a better grasp of the mechanisms for contagion, or *how* depression spreads from one person to another. Several mechanisms have been proposed, included co-rumination among adolescent friends [39], cognitive styles [19], and social norms and reinforcement [21], but the mechanism may depend on the type and closeness of relationship.

Given the increased number of students beginning college with heightened depressive affect, this research has implications for university administrators. As universities put more resources into student mental health services, they might also consider how to include roommates. Not only might they consider reaching out to roommates of students presenting with depressive symptoms, but they could proactively enroll roommate dyads in wellness classes and activities. Given that particularly close roommates might be the most likely to sign up together for such activities, universities could reap a high level of mental health benefits for their students in offering them.

### Constraints on generality

There are several constraints on the generality of the current work. First, the majority of roommate dyads in this study (87%) are women. Although we recruited all incoming first year students at participating universities, women were more likely to respond to recruitment efforts. Because women have higher depression rates, they may be more affected by emotional contagion then men [22], and are more likely to co-ruminate [39]. Future work might focus on multiple mechanisms and larger samples of male dyads. Our sample also reflected the racial and ethnic breakdown of the universities, and as such was 82% white. More work with more ethnically diverse samples is needed to examine whether the current results replicate across different groups.

The current study was conducted across three large public American universities, which may also constrain the generality of our findings. Results might be different at small colleges with smaller classes or greater overlap of peers in multiple classes. Furthermore, at many institutions of higher education, students do not live on campus and may commute from home. For these students, contagion of depressive symptoms and negative effects on academic outcomes may be more likely to occur in familial relationships. In addition, the research was conducted only at American universities. These results may not generalize to universities and roommate relationships outside of the United States. Future research should examine the generalizability of these findings to other types of academic institutions.

Finally, we followed student outcomes for one year, but it may be that over the course of multiple years the effects we observed either multiply or fade. We also had a relatively low number of students who withdrew from courses each semester (9% in Fall, 13% in Spring), limiting our power to find an effect. Also, as with all correlational studies, it may be there are unmeasured variables that help to explain the effects. A study examining depression and academics through graduation would shed more light on the early influences of depression and roommate relationships and allow for greater specificity in predictors.

## Conclusions

The present study provides important information for students and universities as they consider what might happen when students start college–either with heightened depressive symptoms themselves or if they are assigned a roommate with heightened depressive symptoms. The good news is that regardless of depressive symptoms, students can form close friendships with their roommates and succeed academically. However, depression can affect grades and may spread between close roommates. Thus, for students suffering from depressive symptoms it is important to seek help and for universities to have ample help available. This will not only improve one’s own mental health and academic outcomes but may also help their new roommate.

## Supporting information

S1 File Safety monitoring plan. (PDF)

S2 File Analyses replacing dyadic closeness with the geometric mean of dyadic closeness. (PDF)

S1 Table Phase 1 detailed results predicting GPA and course withdrawal. (PDF)

S2 Table Phase 2 detailed results for GPA, course withdrawal, and depressive symptoms contagion. (PDF)

S3 Table Correlations between individual items used to assess dyadic closeness. (PDF)

S1 Data Data set. (SAV)
