# Supplementary material for: Understanding the role of depressive symptoms in academic outcomes: A longitudinal study of college roommates
Source: PLoS One. 2023 Jun 5;18(6):e0286709. doi: 10.1371/journal.pone.0286709 (PMC10241356; doi:10.1371/journal.pone.0286709)
Supplement: S1 File — (PDF) [file pone.0286709.s001.pdf]

Safety Monitoring Plan

Participant responses to all surveys were screened within 48 hours by the project manager. If responses met our criteria for distress (see below), a licensed clinical psychologist contacted participants (by phone) within 24 hours to explain the concern, assess the level of risk and impairment, provide specific resources, and encourage them to follow through with the recommended resources. If the participant did not respond to three attempts at phone calls, the psychologist followed up with resources by e-mail. In addition, letters of support from each university were gathered, such that each counseling center was aware of the study and agreed to the resources given to participants.

Criteria for distress: We established criteria for monitoring participant responses for elevated psychological distress. The first criterion for evaluation was elevated scores on the CES-D. A cutoff score of 16 on the CES-D is generally used to denote mild to moderate levels of depressive symptoms (Radcliff, 1977); however, a score of 16 is generally insufficient to identify clinical levels of depression in college students (Santor, Zuroff, Ramsey, Cervantes, & Palacios, 1995). We used the recommended cutoff score of 27 as an indication of clinical levels of depressive symptoms (Zich, Attkisson, & Greenfield, 1990). If a student met the CES-D cutoff of 27, we then examined the degree of distress and impairment reported using a modified version of the Quality of Life Enjoyment and Satisfaction Questionnaire-Short Form (Q-LES-Q-SF) (Endicott, Nee, Harrison, & Blumenthal, 1993). The Q-LES-Q-SF is widely used among medical, clinical, and psychiatric populations to assess life satisfaction in specific domains and degree of psychological distress. Low scores on the Q-LES-Q-SF are indicative of lower satisfaction and higher distress/impairment in daily functioning. If participants scored a Q-LES-Q-SF score of 57.5 or lower, along with the CES-D of 27 or higher (or a Beck Anxiety Inventory (BAI) score of 26 or higher), he or she was contacted by the clinical psychologist as described above.
