# Supplementary material for: Understanding the role of depressive symptoms in academic outcomes: A longitudinal study of college roommates
Source: PLoS One. 2023 Jun 5;18(6):e0286709. doi: 10.1371/journal.pone.0286709 (PMC10241356; doi:10.1371/journal.pone.0286709)
Supplement: S2 File — (PDF) [file pone.0286709.s002.pdf]

Analyses Replacing Dyadic Closeness with the Geometric Mean of Dyadic Closeness

We conducted Phase 1 and Phase 2 analyses a second time, replacing the dyadic closeness variable with the geometric mean of dyadic closeness. Results did not change in these new analyses.

Phase 1 analyses tested whether students' and roommates' T1 depressive symptoms predicted students' fall and spring GPAs and withdrawals. In our original analyses, dyadic closeness did not moderate any of these effects. When we tested these models a second time including the geometric mean of dyadic closeness as the moderator, results remain unchanged.

Geometric mean of dyadic closeness moderating the links between

- Students' T1 depressive symptoms and students' fall GPA: p = .102, *pseudo* ΔR^2 = .004
- Roommates' T1 depressive symptoms and students' fall GPA: p = .477, *pseudo* ΔR^2 < .001
- Students' T1 depressive symptoms and students' spring GPA: p = .299, *pseudo* ΔR^2 < .001
- Roommates' T1 depressive symptoms and students' spring GPA: p = .200, *pseudo* ΔR^2 < .001

- Students' T1 depressive symptoms and students' fall withdrawals: p = .439
- Roommates' T1 depressive symptoms and students' fall withdrawals: p = .591
- Students' T1 depressive symptoms and students' spring withdrawals: p = .317
- Roommates' T1 depressive symptoms and students' spring withdrawals: p = .728

Phase 2 analyses tested contagion of students' and roommates' GPAs, withdrawals and depressive symptoms at T2 and T3. In our original analyses, dyadic closeness moderated the contagion of GPA in the fall and spring. Dyadic closeness also moderated the contagion of depressive symptoms at the end of the fall and spring semesters but not at the beginning of the fall semester. Dyadic closeness did not moderate the contagion of withdrawals in fall or spring. When we tested these models a second time including the geometric mean of dyadic closeness as the moderator, results remain unchanged. Importantly, for contagion of GPA and depressive symptoms, simple slopes analyses suggest that the patterns of these slopes were similar to those described in the manuscript.

Geometric mean of dyadic closeness moderating

- Contagion of fall GPA: p = .021, *pseudo* ΔR^2 = .01
  - Simple slope for 1sd above the geometric mean of dyadic closeness: b = .31, p < .001
  - Simple slope for 1sd below the geometric mean of dyadic closeness: b = .10, p = .090
- Contagion spring GPA: p = .012, *pseudo* ΔR^2 = .02
  - Simple slope for 1sd above the geometric mean of dyadic closeness: b = .29, p < .001
  - Simple slope for 1sd below the geometric mean of dyadic closeness: b = .07, p = .325

- Contagion of fall withdrawals: p = .348, *pseudo* ΔR^2 = .002
- Contagion spring GPA: p = .401, *pseudo* ΔR^2 = .005

- Contagion of depressive symptoms at the beginning of the fall semester: p = .064, *pseudo* ΔR^2 = .006
- Contagion of depressive symptoms at the end of the fall semester: p = .006, *pseudo* ΔR^2 = .02
    - Simple slope for 1sd above the geometric mean of dyadic closeness: b = .25, $p < .001$
    - Simple slope for 1sd below the geometric mean of dyadic closeness: b = -.03, p = .640
- Contagion of depressive symptoms at the end of the spring semester: p = .038, *pseudo* ΔR^2 = .01
    - Simple slope for 1sd above the geometric mean of dyadic closeness: b = .21, p = .001
    - Simple slope for 1sd below the geometric mean of dyadic closeness: b = .01, p = .859

Phase 3 analyses tested indirect effects of models in which students' T1 depressive symptoms and the contagion of depressive symptoms between roommates within T2 and T3 predicted roommates' GPA and course withdrawals in the fall and spring, respectively. The contagion of depressive symptoms within T2 and T3 was moderated by dyadic closeness. In the manuscript, all of these models showed patterns of conditional mediation. When we replace dyadic closeness with the geometric mean of dyadic closeness, results do not change. We report detailed findings for each model below.

In the model predicting roommates' fall GPA, students' T1 depressive symptoms predicted students' greater T2 depressive symptoms (standardized estimate = .59, 95% CI [.56, .61], $p < .001$). The path from students' T2 depressive symptoms and roommates' T2 depressive symptoms was moderated by dyadic closeness (effect = .08, 95% CI [.05, .11], $p < .001$). Roommates' T2 depressive symptoms, in turn, predicted their lower fall GPA (standardized estimate = -.22, 95% CI [-.24, -.19], $p < .001$). The index of conditional mediation was significant (effect= -.01, 95% CI [-.015, -.007], $p < .001$), such that the indirect effect was significant for dyads who were higher in closeness (effect = -.02, 95% CI [-.03, -.02], $p < .001$) but not for dyads who were lower in closeness (effect = -.00, 95% CI [-.01, .00], p = .233).

In the model predicting roommates' fall withdrawals, students' T1 depressive symptoms predicted students' greater T2 depressive symptoms (standardized estimate = .60, 95% CI [.57, .62], $p < .001$). The path from students' T2 depressive symptoms and roommates' T2 depressive symptoms was moderated by dyadic closeness (effect = .08, 95% CI [.05, .10], $p < .001$). Roommates' T2 depressive symptoms, in turn, predicted their increased fall withdrawals (standardized estimate = .03, 95% CI [.02, .04], $p < .001$). The index of conditional mediation was significant (effect = .001, 95% CI [.001, .002], $p < .001$), such that the indirect effect was significant for dyads who were higher in closeness (effect = .003, 95% CI [.002, .005], $p < .001$) but not for dyads who were lower in closeness (effect = .00, 95% CI [.000, .001], p = .334).

In the model predicting roommates' spring GPA, students' T1 depressive symptoms predicted students' greater T3 depressive symptoms (standardized estimate = .52, 95% CI [.50, .55], p <

.001). The path from students' T3 depressive symptoms and roommates' T3 depressive symptoms was moderated by dyadic closeness (effect = .07, 95% CI [.04, .10], $p < .001$). Roommates' T3 depressive symptoms, in turn, predicted their lower spring GPA (standardized estimate = -.21, 95% CI [-.25, -.18], $p < .001$). The index of conditional mediation was significant (effect = -.01, 95% CI [-.012, -.004], $p < .001$), such that the indirect effect was negative and significant for dyads who were higher in closeness (effect = -.01, 95% CI [-.013,-.00], $p = .042$) but positive and significant for dyads who were lower in closeness (effect = .01, 95% CI [.004, .015], $p = .002$).

In the model predicting roommates' spring withdrawals, students' T1 depressive symptoms predicted students' greater T3 depressive symptoms (standardized estimate = .52, 95% CI [.49, .54, $p < .001$). The path from students' T3 depressive symptoms and roommates' T2 depressive symptoms was moderated by dyadic closeness (effect = .07, 95% CI [.03, .10], $p < .001$). Roommates' T3 depressive symptoms, in turn, predicted their increased spring withdrawals (standardized estimate = .05, 95% CI [.04, .06], $p < .001$). The index of conditional mediation was significant (effect = .002, 95% CI [.001, .003], $p < .001$), such that the indirect effect was positive and significant for dyads who were higher in closeness (effect = .001, 95% CI [.000, .003], $p = .028$) but negative and significant for dyads who were lower in closeness (effect = -.002, 95% CI [-.003, -.001], $p = .002$).
