# Supplementary material for: Understanding the role of depressive symptoms in academic outcomes: A longitudinal study of college roommates
Source: PLoS One. 2023 Jun 5;18(6):e0286709. doi: 10.1371/journal.pone.0286709 (PMC10241356; doi:10.1371/journal.pone.0286709)
Supplement: S1 Table — (PDF) [file pone.0286709.s003.pdf]

Table S1a.

Phase 1 Analyses Predicting GPA: Detailed Results Testing Students' and Roommates' T1 Depressive Symptoms predicting Students' Fall and Spring GPAs.

	DV: Students' Fall GPA					DV: Students' Spring GPA				
	b	*t*(df)	95% *CI*	*p*	*Pseudo* ΔR^2	*b*	*t*(df)	95% *CI*	*p*	*Pseudo* ΔR^2
<u>Main Effects Model</u>										
Students' Standardized Test Scores	.23	4.08 (395.31)	[.12, .34]	<.001		.22	3.96 (388.46)	[.11, .32]	<.001	
Students' T1 Depressive Symptoms	-.19	-3.90 (410.65)	[-.28, -.09]	<.001		-.14	-3.01 (398.95)	[-.24, -.05]	.003	
Roommates' T1 Depressive Symptoms	-.08	-1.81 (409.56)	[-.18, .01]	.072		-.03	-.67 (395.78)	[-.12, .06]	.501	
<u>Tests of Product Terms:</u>										
Model 1:										
Students' Standardized Test Scores	.23	4.18 (382.08)	[.12, .34]	<.001		.22	3.90 (386.84)	[.11, .32]	<.001	
Students' T1 Depressive Symptoms	-.17	-3.55 (408.09)	[-.27, -.08]	<.001		-.15	-3.10 (395.51)	[-.24, -.05]	.002	
Roommates' T1 Depressive Symptoms	-.08	-1.80 (406.80)	[-.18, .01]	.073		-.03	-.70 (393.53)	[-.12, .06]	.485	
Dyadic Closeness	.07	1.36 (223.80)	[-.03, .17]	.176		-.07	-1.33 (226.97)	[-.16, .03]	.186	
Students' T1 Depressive Symptoms * Dyadic Closeness	.08	1.70 (412.99)	[-.01, .17]	.089	<.01	-.04	-.90 (394.70)	[-.14, .05]	.368	<.01
Model 2:										
Students' Standardized Test Scores	.23	4.07 (391.47)	[.12, .34]	<.001		.21	3.90 (387.12)	[.11, .32]	<.001	
Students' T1 Depressive Symptoms	-.18	-3.78 (408.33)	[-.28, -.09]	<.001		-.15	-3.19 (397.55)	[-.25, -.06]	.002	
Roommates' T1 Depressive Symptoms	-.07	-1.56 (407.41)	[-.17, .02]	.119		-.03	-.67 (392.03)	[-.12, .06]	.501	
Dyadic Closeness	.07	1.30 (222.10)	[-.03, .17]	.196		-.06	-1.24 (224.50)	[-.16, .04]	.216	
Roommates' T1 Depressive Symptoms * Dyadic Closeness	.03	.61 (412.98)	[-.06, .12]	.537	<.01	.05	1.22 (399.22)	[-.03, .14]	.224	<.01

Note: All variables are standardized. To test product terms, we added the main effect of closeness and the relevant product term to the original main effects model. All product terms were tested in separate models.

Table S1b.

Phase 1 Analyses Predicting Withdrawal: Detailed Results Testing Students' and Roommates' T1 Depressive Symptoms Predicting Students' Fall and Spring Withdrawal.

	DV: Students' Fall Withdrawals				DV: Students' Spring Withdrawals			
	B	Exp(B)	95% CI for Exp(B)	*p*	B	Exp(B)	95% CI for Exp(B)	*p*
<u>Main Effects Model</u>								
Students' T1 Depressive Symptoms	.47	1.60	[1.15, 2.22]	.005	.38	1.46	[1.14, 1.86]	.002
Roommates' T1 Depressive Symptoms	-.08	.92	[.69, 1.23]	.571	.01	1.01	[.77, 1.33]	.936
<u>Tests of Product Terms:</u>								
Model 1:								
Students' T1 Depressive Symptoms	.47	1.61	[1.15, 2.25]	.006	.34	1.40	[1.09, 1.80]	.008
Roommates' T1 Depressive Symptoms	-.06	.94	[.70, 1.26]	.676	.01	1.01	[.77, 1.33]	.958
Dyadic Closeness	.17	1.18	[.82, 1.71]	.366	-.10	.91	[.69, 1.20]	.502
Students' T1 Depressive Symptoms * Dyadic Closeness	-.12	.89	[.66, 1.20]	.447	-.11	.90	[.73, 1.11]	.323
Model 2:								
Students' T1 Depressive Symptoms	.49	1.63	[1.17, 2.28]	.004	.37	1.44	[1.13, 1.83]	.003
Roommates' T1 Depressive Symptoms	-.08	.92	[.70, 1.23]	.586	-.01	.99	[.75, 1.31]	.955
Dyadic Closeness	.11	1.12	[.81, 1.55]	.5041	-.12	.89	[.68, 1.15]	.356
Roommates' T1 Depressive Symptoms * Dyadic Closeness	-.06	.95	[.74, 1.21]	.648	-.03	.97	[.77, 1.22]	.806

Note: All variables are standardized. To test product terms, we added the main effect of closeness and the relevant product term to the original main effects model. All product terms were tested in separate models.
