# Supplementary material for: Understanding the role of depressive symptoms in academic outcomes: A longitudinal study of college roommates
Source: PLoS One. 2023 Jun 5;18(6):e0286709. doi: 10.1371/journal.pone.0286709 (PMC10241356; doi:10.1371/journal.pone.0286709)
Supplement: S2 Table — (PDF) [file pone.0286709.s004.pdf]

Table S2a

Phase 2 Analyses Predicting GPA: Detailed Results Testing GPA Contagion Within the Fall and Spring Semesters

	DV: Students' Fall GPA					DV: Students' Spring GPA				
	b	*t*(df)	95% *CI*	*p*	*Pseudo* ΔR^2	*b*	*t*(df)	95% *CI*	*p*	*Pseudo* ΔR^2
Main Effects Model										
Students' Standardized Test Scores	.22	3.57 (370)	[.10, .34]	<.001		.23	3.83 (335.93)	[.11, .35]	<.001	
Roommates' Standardized Test Scores	-.03	-.55 (356.54)	[-.16, .09]	.584		-.02	-.29 (310.40)	[-.13, .10]	.776	
Roommates' Fall/Spring GPA	.19	3.79 (333.86)	[.09, .28]	<.001		.20	4.22 (278.48)	[.11, .30]	<.001	
Main Effects Model, Controlling for Students' and Roommates' T1 Depressive Symptoms										
Students' Standardized Test Scores	.21	3.45 (368.94)	[.09, .33]	.001		.22	3.71 (337.96)	[.10, .34]	<.001	
Roommates' Standardized Test Scores	-.04	-.68 (356.27)	[-.16, .08]	.500		-.02	-.35 (312.67)	[-.14, .10]	.727	
Students' T1 Depressive Symptoms	-.18	-3.64 (373.98)	[-.28, -.08]	<.001		-.14	-2.69 (351.99)	[-.24, -.04]	.007	
Roommates' T1 Depressive Symptoms	-.06	-1.26 (343.03)	[-.16, .04]	.209		-.03	-.68 (299.98)	[-.13, .06]	.497	
Roommates' Fall/Spring GPA	.16	3.20 (335.46)	[.06, .26]	.001		.19	3.97 (286.83)	[.10, .29]	<.001	
Tests of Product Terms:										
Students' Standardized Test Scores	.23	3.73 (367.92)	[.11, .35]	<.001		.23	3.87 (334.56)	[.11, .35]	<.001	
Roommates' Standardized Test Scores	-.04	-.66 (354.79)	[-.16, .08]	.508		-.02	-.35 (309.33)	[-.14, .10]	.730	
Roommates' Fall/Spring GPA	.20	4.05 (334.09)	[.10, .30]	<.001		.18	3.74 (284.68)	[.09, .28]	<.001	
Dyadic Closeness	.09	1.82 (364.33)	[-.01 .19]	.070		-.00	-.08 (238.84)	[-.10, .09]	.937	
Roommates' Fall/Spring GPA * Dyadic Closeness	.10	2.22 (313.33)	[.01, .19]	.028	.01	.10	2.43 (276.78)	[.02, .19]	.016	.02

Note: All variables are standardized. To test product terms, we added the main effect of closeness and the relevant product term to the original main effects model. All product terms were tested in separate models.

Table S2b

Phase 2 Analyses Predicting Withdrawal: Detailed Results Testing Withdrawal Contagion Within the Fall and Spring Semesters

	DV: Students' Fall Withdrawal					DV: Students' Spring Withdrawal				
	B	Exp(B)	95% CI for Exp(B)	*p*	*Pseudo* ΔR^2	B	Exp(B)	95% CI for Exp(B)	*p*	*Pseudo* ΔR^2
<u>Main Effects Model, Controlling for Students' T1 Depressive Symptoms</u>										
Students' T1 Depressive Symptoms	.49	1.63	[1.21, 2.20]	.001		.40	1.49	[1.49, 1.93]	.003	
Roommates' Fall/Spring Withdrawal	1.10	3.00	[1.26, 7.10]	.013		-.34	.71	[.29, 1.75]	.461	
<u>Tests of Product Terms:</u>										
Roommates' Fall/Spring Withdrawal	1.02	1.25	[1.17, 6.51]	.020		-.47	.63	[.23, 1.70]	.356	
Dyadic Closeness	.23	.70	[.87, 1.82]	.232		-.04	.96	[.72, 1.29]	.799	
Roommates' Fall/Spring Withdrawal * Dyadic Closeness	-.36	.08	[.27, 1.83]	.464	<.01	-.35	.70	[.29, 1.72]	.439	<.01

Note: All variables are standardized. To test product terms, we added the main effect of closeness and the relevant product term to the original main effects model. All product terms were tested in separate models.

Table S2c

Phase 2 Analyses: Detailed Results Testing Closeness Moderating Contagion of Depressive Symptoms at T1, T2, and T3

	DV: Students' Depressive Symptoms				DV: Students' Depressive Symptoms				
	b	*t*(df)	95% *CI*	*p*	*b*	*t*(df)	95% *CI*	*p*	*Pseudo* ΔR^2
T1 Contagion of Depressive Symptoms									
Roommates' T1 Depressive Symptoms	.03	.59 (428.81)	[-.06, .11]	.593	.02	.48 (431.38)	[-.07, .11]	.631	
Dyadic Closeness					-.12	-2.67 (471.34)	[-.21, -.03]	.008	
Roommates' T1 Depressive Symptoms * Dyadic Closeness					.07	1.75 (447)	[-.01, .16]	.081	<.01
T2 Contagion of Depressive Symptoms									
Roommates' T2 Depressive Symptoms	.12	2.61 (334.12)	[.03, .21]	.009	.11	2.35 (335.05)	[.02, .20]	.019	
Dyadic Closeness					.01	.12 (383.43)	[-.10, .11]	.905	
Roommates' T2 Depressive Symptoms * Dyadic Closeness					.14	2.70 (340.31)	[.04, .24]	.007	.02
T3 Contagion of Depressive Symptoms									
Roommates' T3 Depressive Symptoms	.12	2.48 (380.35)	[.02, .22]	.014	.11	2.34 (388.03)	[.02, .21]	.020	
Dyadic Closeness					.04	.75 (400.61)	[-.06, .14]	.454	
Roommates' T3 Depressive Symptoms * Dyadic Closeness					.10	2.13 (362.53)	[.01, .20]	.034	<.01

Note: All variables are standardized. Complete results for main effects are reported in the manuscript. To test product terms, we added the main effect of closeness and the relevant product term to the original main effects model. All product terms were tested in separate models.
