# Supplementary material for: Understanding the role of depressive symptoms in academic outcomes: A longitudinal study of college roommates
Source: PLoS One. 2023 Jun 5;18(6):e0286709. doi: 10.1371/journal.pone.0286709 (PMC10241356; doi:10.1371/journal.pone.0286709)
Supplement: S3 Table — (PDF) [file pone.0286709.s005.pdf]

S3 Table

Correlations Between Individual Items Used to Assess Dyadic Closeness

	1.	2.	3.	4.	5.
1. Close1_A					
2. Close2_A	.74***				
3. Close3_A	.76***	.70***			
4. Close1_P	.54***	.42***	.53***		
5. Close2_P	.42***	.33***	.41***	.74***	
6. Close3_P	.53***	.41***	.51***	.76***	.70***

Note. *** $p<.001$. Close1_A and Close1_P: "How close do you feel to your roommate?" Close2_A and Close2_P: "Relative to all your other relationships (both same and opposite sex), how would you characterize your relationship with your roommate?" Close3_A and Close3_P: "Relative to what you know about other people's roommate relationships, how would you characterize your relationship with your roommate?"
